# Antimicrobial and Cytotoxic Potential of Eucalyptus Essential Oil-Based Nanoemulsions for Mouthwashes Application

**DOI:** 10.3390/antibiotics13100942

**Published:** 2024-10-08

**Authors:** Dione Glauco Batista, William Gustavo Sganzerla, Lysa Ribeiro da Silva, Yasmin Gabriele Schmitt Vieira, Aline R. Almeida, Diogo Dominguini, Luciane Ceretta, Adriana Castro Pinheiro, Fabiano Cleber Bertoldi, Daniela Becker, Dachamir Hotza, Michael Ramos Nunes, Cleonice Gonçalves da Rosa, Anelise Viapiana Masiero

**Affiliations:** 1 Multi-User Laboratory, Graduate Program in Environment and Health, Planalto Catarinense University, Lages 88509-900, SC, Brazil; dioneglaucob@uniplaclages.edu.br (D.G.B.); lysaribeiro@uniplaclages.edu.br (L.R.d.S.); yasmin.schmitt@uniplaclages.edu.br (Y.G.S.V.); cleo.rosa@uniplaclages.edu.br (C.G.d.R.); anemasiero@uniplaclages.edu.br (A.V.M.); 2 School of Food Engineering (FEA), University of Campinas (UNICAMP), Campinas 13083-970, SP, Brazil; 3 Laboratory of Plasmas, Films, and Surfaces, Santa Catarina State University (UDESC), Joinville 89219-710, SC, Brazil; alinerosaufpr@gmail.com (A.R.A.); daniela.becker@udesc.br (D.B.); 4 Laboratory of Experimental Pathophysiology, Graduate Program in Health Sciences, University of Southern Santa Catarina (UNESC), Criciúma 88806-000, SC, Brazil; diogo_dominguini@unesc.net; 5 Graduate Program in Collective Health, University of Southern Santa Catarina (UNESC), Criciúma 88806-000, SC, Brazil; luk@unesc.net; 6 Center of Chemical, Pharmaceuticals, and Food Sciences, Federal University of Pelotas, Pelotas 96010-610, RS, Brazil; adrianacastropinheiro@gmail.com; 7 Agricultural Research and Rural Extension Company of Santa Catarina (EPAGRI), Itajaí 88318-112, SC, Brazil; fabianobertoldi@epagri.sc.gov.br; 8 Graduate Program in Chemical Engineering (PosENQ), Federal University of Santa Catarina (UFSC), Florianópolis 88040-900, SC, Brazil; d.hotza@ufsc.br (D.H.); michael.nunes@ifsc.edu.br (M.R.N.); 9 Department of Chemical and Food Engineering (EQA), Federal University of Santa Catarina (UFSC), Florianópolis 88040-900, SC, Brazil; 10 Federal Institute of Santa Catarina, Lages 88506-400, SC, Brazil; 11 Department of Endodontics, College of Dentistry and Dental Clinics, University of Iowa, Iowa City, IA 52242, USA

**Keywords:** nanotechnology, preventive dentistry, antioxidant, bioactive compounds

## Abstract

**Objective:** An eucalyptus essential oil-based nanoemulsion was produced and evaluated for its antimicrobial properties against *Streptococcus mutans* and its cytotoxicity in the surface mucous cells of rabbits. **Methods:** The essential oil-based nanoemulsion was synthesized with two species of eucalyptus—*Eucalyptus citriodora* and *Eucalyptus globulus*—followed by physicochemical characterization and the determination of antimicrobial activity and cell viability. Subsequently, the mouthwash formulations (fluoride and fluoride-free) were functionalized with the nanoemulsion, and their in vitro antimicrobial actions were evaluated against *S. mutans*. **Results:** The nanoemulsion presented an average particle size of around 100 nm, a polydispersity index close to 0.3, a zeta potential between −19 and −30 mV, a pH close to 7, a spherical shape, and a cell viability above 50%. The antimicrobial activity analysis showed that the nanoemulsion was effective in the control of *S. mutans.* The mouthwashes functionalized with the nanoemulsion also presented bacteriostatic and bactericidal properties. **Conclusions:** The bio-based material produced with eucalyptus essential oil presented adequate physicochemical characteristics, with the potential to be used as an innovative material in preventive dentistry, contributing to the maintenance of oral and systemic health.

## 1. Introduction

Dental caries is a diet-modulated, dynamic, multifactorial, non-transmissible disease, resulting in the loss of organic and mineral tissue [1]. Dental caries are determined by biological, behavioral, psychosocial, and environmental factors [2], and approximately 2.3 billion people around the world have caries in their permanent teeth [3]. In addition, periodontal disease is a chronic non-communicable disease, considered an important public health problem, with prevalence in around 20 to 50% of the global population, with no difference between developed and developing countries [4]. It may vary according to social context [5], but the demographic transition and aging population may be related to its increasing prevalence, especially in developed countries [4].

Biofilms are essential components involved in the development of caries and periodontal disease. Therefore, knowledge of their composition and microbial interactions is critical to establish preventive and therapeutic measures [6]. Although brushing, flossing, and water fluoridation are considered effective preventive methods [7], other alternative methods, for example, mouthwashes, can be used as a complement to the mechanical action and chemical control of microbial plaque for the treatment of inflammation and infections in the oral cavity [8]. Mouthwashes are among the most used vehicles to evaluate antimicrobial compounds, since they prevent the adhesion of microorganisms, which corresponds to the initial stage of biofilm formation [9]. Some of the most studied active agents are chlorhexidine gluconate, essential oil, and cetylpyridinum chloride [10].

Essential oils are volatile products extracted from aromatic plants’ leaves, bark, and fruits, and their medicinal properties have been known since ancient times [11]. Eucalyptus essential oil consists of monoterpenes, hydrocarbons, alcohols, ethers, ketones, and lactones, among other compounds [12]. These compounds are responsible for the antimicrobial action of this essential oil [13]. The application of essential oils in dentistry can occur in different ways—in toothpaste, mouthwashes, and gels—mainly because of their antiseptic properties, which characterize them as an alternative to chlorhexidine for plaque and gingivitis reduction [8,14,15]. Although chlorhexidine is an effective antimicrobial agent, it causes side effects such as tooth staining, oral mucosa erosion, elimination of desirable bacteria, development of antimicrobial-resistant strains, increased formation of supragingival calculi, taste impairments, burning sensation, etc., preventing its long-term use [16].

Considering the above-mentioned side effects, using nanoemulsions as carriers of natural substances such as essential oils seems to be a promising strategy. Nanoemulsion formulations have been recently proposed in the literature for their antimicrobial activity against *Streptococcus mutans* [17,18]. Nanoemulsions are systems dispersed between two immiscible liquids, whose droplets have a mean size of less than 200 nm [19]. To stabilize the aqueous phase and oil phase, surfactants are used to obtain homogeneous and stable samples [19]. Nanoemulsion formulations offer several advantages, for example, delivery of drugs such as biological or diagnostic agents. One of the most important applications of nanoemulsions is to mask the unpleasant taste of oily liquids, protecting drugs that are susceptible to hydrolysis and oxidation, as well as prolonging their action [20].

Currently, there is a limited number of studies evaluating essential oil-based nanoemulsions for application in mouthwashes. Therefore, the present work developed an eucalyptus essential oil-based nanoemulsion and evaluated the physicochemical properties, antimicrobial activity, and cellular viability for use in mouthwashes.

## 2. Results and Discussion

### 2.1. Chemical Characterization of Eucalyptus Essential Oil

Essential oils are secondary plant metabolites, and their chemical composition may vary depending on the species, environmental conditions, plant parts extracted, time of year, and other factors [21]. The major compound in the essential oil of *Eucalyptus globulus* is 1,8-cineole, which accounts for 87.21%, while in *Eucalyptus citriodora*, citronellal accounts for 90.26%, as shown in Table 1. 

The essential oil of *Eucalyptus* species has been widely studied for its antimicrobial [21,22,23,24,25] and anti-inflammatory properties [26]. The antimicrobial activity of eucalyptus essential oil is associated with the synergistic effects between its primary and secondary components, rather than the isolated concentration of a single component [27]. Its main components, such as 1,8-cineole, citronellol, α-pinene, β-pinene, and limonene, demonstrate toxicity against a variety of microorganisms, including bacteria, viruses, and fungi [28,29]. Studies show that eucalyptus essential oil does not affect microbial DNA, unlike other compounds such as cajanol and monoterpene indole alkaloids, which can damage the DNA of microorganisms [13,21]. The volatile compounds of the essential oil exhibit antimicrobial activity both through direct absorption by the microorganisms and indirectly via the medium that absorbs the volatiles.

The anti-inflammatory effect of eucalyptus essential oil acts as an immunoregulatory agent, stimulating the innate immune response mediated by cells. This suggests its potential as an adjuvant in the immunosuppression of infectious diseases [26]. Traditionally, Eucalyptus is used to treat infections, colds, flu, sore throats, bronchitis, pneumonia, as well as pain and neuralgia [30].

### 2.2. Physicochemical Characterization of Nanoemulsions 

#### 2.2.1. Particle Size and Polydispersity Index

In this study, the nanoemulsions of NanoEE-Globulus showed smaller droplet sizes when compared to the nanoemulsions of NanoEE-Citriodora. Both nanoemulsions showed physicochemical stability concerning the parameters of droplet size and polydispersity index, as shown in Table 2.

In this study, the samples of NanoEE-Globulus and NanoEE-Citriodora showed a mean size around 100 nm, with PDI values close to 0.3. The size of the nanoemulsion droplets depends on factors such as the nature and concentration of the surfactant in the aqueous and oil phases, the nature of the essential oil being used, the solvent polarity, and the nature and the proportion of the aqueous and oil phases [21]. Small droplet diameters, ranging from 100 to 200 nm, as in the results found in the present study, are fundamental for the absorption and in vivo distribution of suspensions [23]. In addition, when considering the development of a nanoemulsion for therapeutic purposes, the determining points for the release of the active agent are droplet size and uniformity [24]. 

The larger the PDI, the larger the particle size range and, consequently, the more heterogeneous the droplet size is in the nanoemulsion [25]. PDI results below 0.3 (as in the present study) show a narrow distribution of size and good homogeneity of the nanoemulsion [22]. The lower the value found for PDI ≤ 0.2, the higher the degree of homogeneity in droplet size, which is an important fact when considering the possibility of the future development of mouthwash because if the system remains with homogeneous droplet size, the product is likely to have greater physicochemical stability [22]. The low values obtained for span corroborate the PDI values, showing a narrow particle size distribution.

#### 2.2.2. Zeta Potential and pH

In the present study, the nanoemulsions of NanoEE-Globulus and NanoEE-Citriodora have zeta potential values ranging from −19 to −30 mV and pH values close to 7, tending to be neutral. The surfactant polysorbate 80 has an apolar part that interacts with the oil and a polar part outside the nanoemulsion, which exposes the negative charge to the nanoemulsion, resulting in a negative surface density, owing to the presence of oxygen atoms in the molecule [22].

The zeta potential measurements depend mainly on the stabilizing agents and the pH of the medium [26]. The zeta potential reflects the surface load of the nanoemulsions and indicates the stability of a nanoemulsion, according to the repulsive forces between the droplets and the potential changes on the droplet surface [27]. Also, it evaluates the capacity that particles can remain in suspension, that is, without aggregation or sedimentation [28].

In general, the zeta potential evaluates nanoemulsion stability; when using blocks of anionic surfactants, such as polysorbate 80 (Tween 80) and sorbitan monooleate (Span 80), as in the present study, dispersion stability is verified by steric impediment, avoiding the coalescence of particles and allowing physicochemical stability [29]. In addition, the absence of electrostatic repulsion is also confirmed by the pH results, with a tendency to be neutral [30]. When there is additional steric stabilization (Tween 80, in this case), values around 20 mV are already sufficient to maintain the stability of the colloidal system [27,31].

#### 2.2.3. Transmission Electron Microscopy

**Figure 1:**
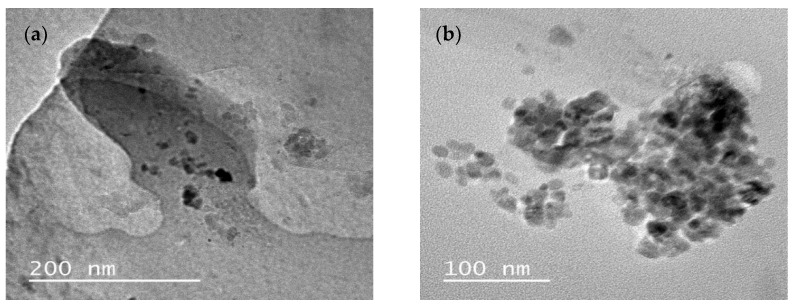
Morphology of nanoemulsions evaluated by transmission electron microscopy: (**a**) NanoEE-Citriodora and (**b**) NanoEE-Globulus.

The micrographs obtained by transmission electron microscopy show the morphology of the particles, which have a spherical shape (Figure 1), and a different size than the one determined by dynamic light scattering (DLS).

The microscopic analysis provides an accurate evaluation of the size and shape of the droplets; however, the variation in particle size between the DLS and TEM techniques can be explained by the preparation of the sample for the reading of the microscope, which may have modified the surface area to be analyzed [32,33]. Sample preparation for TEM measurements requires techniques such as solvent evaporation, flattening of particles in the *grid*, and fewer particles visualized by microscopy when compared to the Light Scattering Technique [26,31,34]. 

#### 2.2.4. Antimicrobial Activity Tests: Inhibitory Concentration of Nanoemulsions

The broth microdilution test was used to determine the MIC of the nanoemulsions. Turbidity was the parameter used to verify the growth of the microorganism in the microplate wells. To this end, the MIC was the lowest concentration among those tested, and there was no turbidity in the growth medium. Both nanoemulsions, NanoEE-Globulus and NanoEE-Citriodora, were effective in controlling *S. mutans* (Table 3). The sample of NanoEE-Globulus presented an MIC of 4% while that of NanoEE-Citriodora showed an MIC of 6% against this Gram-positive bacterium, revealing that the samples present inhibitory activity even at the lowest concentration tested. 

The antimicrobial action mechanisms of eucalyptus essential oil consist of denaturation of the action of bacterial proteins, inactivation of microbial enzymes, alteration of membrane permeability of Gram-negative bacteria, and chelation of cation ions present in bacterial cytoplasm [35]. In a study evaluating antimicrobial action, eucalyptus essential oil demonstrated effectiveness against bacteria present in the oral cavity *(S. aureus* and *E. faecalis*), with efficacy similar to that of 0.12% chlorhexidine, which is considered the gold standard for mouthwashes [36].

Harkat Madouri et al. [37] conducted a literature review that evaluated the effect of *Eucalyptus globulus labill* essential oil on microorganisms related to periodontal disease and reported that the following MICs are, respectively, needed to inhibit the bacteria *P. gingivalis* (0.28 mg mL^−1^), *F. nucleatum* (1.14 mg mL^−1^), and *A. actinomycetemprincipans* (AA) (9.13 mg mL^−1^). The result highlights the highest resistance of AA.

There is a limitation in the literature when comparing the results of the present study; in addition to the limited number of studies that have tested essential oils for application in mouthwashes, there is a diversity of presentations, methodologies, and oils. There is a predominance of in vitro studies in particular [18,22,38,39]; one study studied the in vitro and in vivo phases in animals [40], and one was a randomized double-blind clinical trial [14].

In a recent study, Karnjana et al. [41] found that the ethanolic extracts of *Streblus asper*, *Cymbopogon citratus*, and *Syzygium aromaticume* can be natural agents with multiple actions on *S. mutans*. Changes in the bacterial cell walls occurred after treatment with the ethanol extracts, increasing hydrophobia and decreasing the formation of bacterial biofilms for 24 h. The authors reported that the extracts were also used for the green synthesis of silver nanoparticles, and the results were also satisfactory.

#### 2.2.5. Nanoemulsion Cytotoxicity and Cell Viability Assays

**Figure 2:**
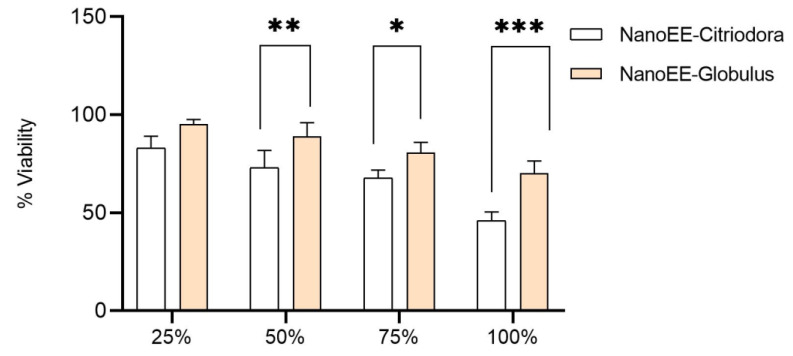
Survival of oral mucosa cells against nanoemulsion samples. The *p* values: * *p* < 0.05; ** *p* < 0.005 and *** *p* < 0.001.

The results for cell viability indicated that nanoemulsions of NanoEE-Globulus showed higher cell viability compared to the NanoEE-Citriodora samples. Specifically, the nanoemulsions at the concentration of 100% maintained a cell viability greater than 50%, suggesting that these nanoemulsions do not have a significant cytotoxic potential (Figure 2). This aspect is essential for using them in products such as mouthwashes.

The difference in cell viability between the nanoemulsions of the NanoEE-Globulus and NanoEE-Citriodora samples can be attributed to the distinct chemical properties of the essential oils involved. Eucalyptus globulus contains 1,8-cineole, a compound that has been associated with a relatively high safety profile for cells at high concentrations. This may explain the result for higher cellular viability, suggesting that NanoEE-Globulus is less toxic to cells.

In contrast, the major component of *Eucalyptus citriodora* is citronellal, which despite its effective antimicrobial properties, may have a more pronounced impact on cell viability, resulting in lower viability compared to NanoEE-Globulus.

The observation that nanoemulsions, even at a concentration of 100%, maintained a cell viability of more than 50% is particularly relevant for applications in products such as mouthwashes. The absence of a cytotoxic potential at high concentrations is a positive point, as it ensures that the product is safe for prolonged use and in direct contact with oral tissues.

Cellular safety is an important factor for the formulation of oral care products because the combination of antimicrobial efficacy and low toxicity is essential. Nanoemulsions of NanoEE-globulus, with their higher cell viability and absence of cytotoxicity at high concentrations, offer a promising profile to be used in mouthwashes and other oral care products. On the other hand, formulations containing NanoEE-Citriodora may need adjustments in their concentration or formulation to ensure similar safety, which is a point to be considered for future applications and product development.

#### 2.2.6. Antimicrobial Activity Assays: Inhibitory Concentration of Mouthwashes Functionalized with Nanoemulsions

**Figure 3:**
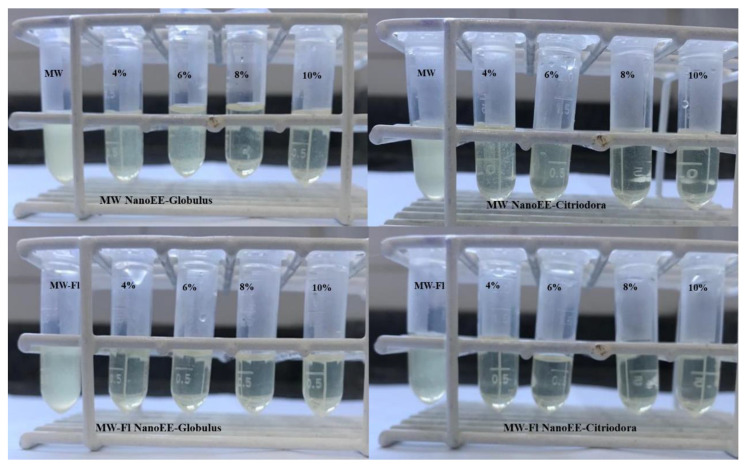
Bacteriostatic effect of mouthwashes functionalized with nanoemulsions.

Fluoride mouthwashes showed a bacteriostatic effect against *S. mutans* in all study concentrations, while fluoride-free mouthwashes showed this effect in concentrations above 8%, as shown in Figure 3.

The antimicrobial action of nanoemulsions at different concentrations was confirmed by comparing them with the control sample containing only the mouthwash. In addition to the bacteriostatic effect, the bactericidal effect was also confirmed in samples that did not present turbidity. The formulation of oral mouthwashes has compounds such as sodium fluoride, which have antimicrobial activity to eliminate harmful oral microorganisms, thus contributing to the prevention of future dental lesions, gingivitis, and periodontitis.

In this study, eucalyptus nanoemulsions demonstrated a synergistic effect with sodium fluoride. When applied topically, such as in toothpastes, gels, or fluoridated solutions, fluoride provides direct and immediate protection [42]. Fluoride efficacy is even greater when combined with good oral hygiene, which includes regular toothbrushing, flossing, and periodic visits to the dentist. This ensures that teeth are less susceptible to the development of cavities and other oral diseases. The mechanism of action against fluoride is mainly associated with its influence on the mineralization of teeth and the process of remineralization, in addition to its impact on plaque bacteria, which cause acidification and demineralization [43].

Fluoride can affect bacterial metabolism in several ways. One of them is acting directly as an enzyme inhibitor, for example, inhibiting the glycolytic enolase enzyme. Another form of action involves the formation of metal–fluoride complexes, such as AlF_4_^−^, which are responsible for inhibiting F-ATPases, which are enzymes that translocate protons. These complexes mimic phosphate, forming complexes with ADP in enzyme reaction centers [43].

However, the most relevant actions of fluoride for reducing plaque cariogenicity are related to its weak acid character. Fluoride increases the permeability of the bacterial membrane to protons, compromising the functioning of F-ATPases in proton export. This leads to cytoplasmic acidification and inhibition of glycolytic enzymes, reducing acid tolerance in bacteria. Fluoride is particularly effective in acidic environments; for example, in acidic conditions in the cariogenic plaque, concentrations as low as 0.1 mm of fluoride can completely stop glycolysis in intact *Streptococcus mutans* cells [44].

In general, the anticaries actions of fluoride are complex, involving effects on both the bacteria and mineral phases of plaque formation. Its antibacterial properties are complex but predominantly influenced by its weak acid character [44].

In synergy with fluoride, eucalyptus essential oil also presents an antimicrobial action. The antimicrobial action of eucalyptus essential oil can be attributed to different compounds, which vary according to the species of eucalyptus and the cultivar. In the case of the species *Eucalyptus globulus*, the main compound responsible for antimicrobial activity is 1,8-cineole (also known as eucalyptol). Studies such as that of Goldbeck [45] indicate that 1,8-cineole can represent up to 71% of essential oil, while other studies, such as the one of Salem (2018) [46], mention a concentration of approximately 13.23%. This variation can be the result of differences in the origin of the samples or in the methods of analysis.

For the species *Eucalyptus citriodora*, the main compound is citronellal, which can account for to 72.7% of the essential oil. Citronellal has antimicrobial properties and is also known for its insect repellent effect. Therefore, the antimicrobial efficacy of eucalyptus essential oil can depend greatly on the specific profile of the compounds present, which varies with species and cultivar.

The study carried out by Choi [47] showed that essential oils containing aldehydes as major compounds exhibit considerable antimicrobial activity against strains of *S. mutans*. The work of Goldbeck et al. [45] showed that the essential oil of *E. globulus* with a high concentration of 1–8 cineole caused the microbial death of strains of *S. mutans*. According to the authors, the antibacterial activity of these compounds is linked to an increase in the permeability of the bacterial membrane and the consequent loss of its cellular elements, which leads to cell collapse.

In the future, mouthwashes are expected to advance to the point of being highly specialized, with action against specific pathogenic bacteria, preventing other beneficial bacteria from the oral microbiome from being affected, thus promoting the balance of such microbiome. In addition, they could modulate the immune response of the host, helping the body fight infections more effectively and even preventing inflammation. These advances would bring significant benefits to oral health, especially for people who have difficulty performing adequate oral hygiene or for those with medical conditions that affect oral health.

## 3. Materials and Methods

### 3.1. Extraction and Characterization of Essential Oils

The essential oil was extracted from the leaves of *Eucalyptus citriodora* and *Eucalyptus globulus* through steam distillation using a Clevenger-type apparatus. The chemical characterization of the essential oils was determined by Gas Chromatography coupled with Mass Spectrometry (GC/MS) using a Shimadzu model GCMS-QP2010 (Shimadzu Corp., Columbia, MD, USA). A ZB-5MS capillary column (30 m × 0.25 mm × 0.25 µm film) (Phenomenex, Torrance, CA, USA) was used. The injector temperature was set to 250 °C, and the helium carrier gas flow rate was 1.0 mL/min. The chromatographic oven was optimized with an initial temperature of 60 °C held for 4 min and then ramped to 210 °C, where it was held for 6 min, totaling a 35 min chromatographic run. The essential oil samples were diluted 200 times in analytical-grade hexane before injection into the GC/MS. Quantification was performed by normalizing the peak area (%) of each chemical constituent, with the total area being the sum of all peak areas in the chromatogram (100%).

Identification by GC-MS was based on a comparison of the mass spectra with National Institute of Standards and Technology—NIST MS library data and also a comparison of the retention indices calculated with literature values [48]. In some cases, the comparison was made with commercial standards injected under the same conditions. Retention indices were calculated according to the method of Van den Dool and Kratz [49] using n-alkane standards (C7–C30) under the same chromatographic conditions as the essential oil samples.

### 3.2. Production of Nanoemulsions

The nanoemulsions were produced with eucalyptus essential oil (*n* = 3) from two different cultivars—*E. citriodora* and *E. globulus,* referred to as NanoEE-Citriodora and NanoEE-Globulus—via emulsification and high-speed stirring with Ultra Turrax^®^ equipment (Thermo Fisher Scientific Inc., Waltham, MA, United States) [22]. The oil phase of the nanoemulsion was composed of 5% essential oil and 2% sorbitan monooleate (Span 80). The aqueous phase was composed of 2% polysorbate 80 (Tween 80) and 25 mL of ultrapure water. Both phases were previously solubilized with a stirrer. The oil nanoemulsion was poured into the aqueous nanoemulsion under stirring at 10,000 rpm, and the stirring speed was increased to 17,000 rpm and maintained for 30 min at a controlled temperature in an ice bath.

### 3.3. Physicochemical and Morphological Characterization of Eucalyptus Essential Oil Nanoemulsions

The physicochemical characterization of eucalyptus essential oil nanoemulsions (NanoEE-Globulus and NanoEE-Citriodora) was established by the parameters average particle size (Z-ave), zeta potential (*ζ*-mV), transmission electron microscopy (TEM), and hydrogenionic potential (pH).

The particle size (nm), polydispersity index, and zeta potential (*ζ*-mV) of the nanoemulsions were determined by dynamic light scattering (DLS) using Zetasizer Nano Series dispersing equipment (Malvern Instruments, Malvern, United Kingdom). The nanoemulsion samples and the control sample were diluted appropriately with Milli-Q^®^ ultrapure water, and measurements were performed at 25 °C at an angle of 173° in triplicate (*n* = 3). For the measurements, the samples were placed in an electrophoresis cell.

The morphology of the nanoemulsions was evaluated by TEM using a JEOL microscope JEM-1011 (Tokyo, Japan) operating at 70 kV. The solutions containing the nanoemulsions and control sample were previously diluted in Milli-^Q®^ ultrapure water, and about 5 µL of each sample was deposited on carbon-coated copper grids (200 mesh). After drying at room temperature, the grids were observed in the microscope.

The pH was determined in a benchtop pH meter using electrodes selective for H^+^ ions. The electrodes were dipped into the nanoemulsion samples for pH reading, and the data were recorded. 

### 3.4. Antimicrobial Evaluation of Nanoemulsions

The minimum inhibitory concentration (MIC) and minimum bactericidal concentration (MBC) were evaluated with the eucalyptus essential oil nanoemulsion, and the control sample was assessed with chlorhexidine (positive control), namely NanoEE-Globulus NanoEE-Citriodora, Chlorhexidine (positive control), and water (negative control).

The in vitro antibacterial activity was determined on microplates using the standard microdilution method adapted from the Clinical and Laboratory Standards Institute (CLSI) (document NCCLS M7-A9, 2012) [50]. Nanoemulsions of NanoEE-Globulus and NanoEE-Citriodora were diluted in tryptic soy broth (TSB), resulting in concentrations of 4, 6, 8, and 10%. Then, 5 μL of bacterial culture (10^5^ CFU/mL) was inoculated at different concentrations, totaling a final volume of 1 mL. Strains of *S. mutans* (ATCC-25175) (gram-positive) were used in the tests. Negative control wells were prepared by replacing the samples with sterile ultrapure water without the bacterial inoculum and positive control wells, by replacing the samples with chlorhexidine and the bacterial inoculum. 

The plates were incubated at 35 °C for 16–20 h, in an anaerobic jar. Bacterial growth inhibition was determined by turbidity in comparison to the control wells. The wells containing a medium that did not present turbidity were replicated in nutrient agar plates and incubated at 37 °C for 24 h to classify them as bactericidal or bacteriostatic.

### 3.5. Cytotoxicity and Cell Viability Test

Initially, the surface mucous cells of rabbits were grown in Dulbecco’s Modified Eagle Medium (DMEM) with high glucose content supplemented with 10% fetal bovine serum, 100 units/mL of penicillin, and 100 μg/mL of streptomycin. They were kept in a moist environment with 5% CO_2_/95% air at 37 °C. A single dose of nanoemulsions (NanoEE-Globulus and NanoEE-Citriodora) was added at concentrations of 25, 50, 75, and 100 μg/mL. The viability of the surface mucous cell cells was determined using an MTT assay (0.5 mg/mL) and trypan blue exclusion (TBE). The cells were grown at a density of 10,000 cells/well in 96-well plates. The cells were stained with 20 μL MTT stock solution (5 mg/mL) for each well for 4 h. After that, the cells were dissolved with DMSO, and the optical density was determined at 490 nm.

To evaluate cell viability, the percentage of viable cells in comparison to that of the control was calculated using the absorbance values relative to the ratio between the cells exposed to treatment (*Absorbance sample*) for absorbance of the cell-free culture medium (*Absorbance control*), as indicated in Equation (1)

(1) $$\mathrm{Cell}\;\mathrm{viability}\left(\%\right)=\frac{\mathrm{Absorbance}\;\mathrm{sample}}{\mathrm{Absorbance}\;\mathrm{control}}\times \;100$$

### 3.6. Application of Nanoemulsions in Mouthwashes

Each nanoemulsion of NanoEE-Globulus and NanoEE-Citriodora was added in a 1:1 ratio in two mouthwash bases: one formulation with fluoride and one without it. The fluoride mouthwash formulation was composed of nipagin 0.02%, glycerin 5%, sorbitol 5%, and 0.2% sodium fluoride, while the other formulation was composed in the same concentrations but with the absence of sodium fluoride. Subsequently, antimicrobial activity was evaluated (Section 2.2.4) for the *S. mutans* strain (ATCC-25175), in the following samples: MW, fluoride-free mouthwash; MW NanoEE-Globulus, fluoride-free mouthwash + NanoEE-Globulus; MW NanoEE-Citriodora, fluoride-free mouthwash + NanoEE-Citriodora; MW-Fl, fluoride mouthwash; MW-Fl NanoEE-Globulus, fluoride mouthwash + NanoEE-Globulus; and MW-Fl NanoEE-Citriodora, fluoride mouthwash + NanoEE-Citriodora.

### 3.7. Statistical Analysis

The data were expressed as means and standard deviation of the determinations carried out in triplicate. The results underwent an analysis of variance, and the means were compared by ANOVA and Tukey’s test with a significance level of 5%, using the Statistica software version 10.0 (StatSoft, Tulsa, OK, United States).

## 4. Conclusions

In this study, the major compound in the essential oil of *Eucalyptus globulus* is 1,8-cineole, which accounts for 87.21%, while in *Eucalyptus citriodora*, citronellal accounted for 90.26%. For physicochemical characteristics, the NanoEE-Globulus and NanoEE-Citriodora samples presented a mean size around 100 nm, a polydispersity index close to 0.3, a zeta potential between −19 and −30, and pH values close to 7. When the antimicrobial activity was evaluated, the NanoEE-Globulus sample showed an MIC of 4% and the NanoEE-citriodora one presented a MIC of 6% against *S. mutans*. Fluoride mouthwashes showed a bacteriostatic effect against *S. mutans* in all study concentrations, while fluoride-free mouthwashes showed this effect in nanoparticle concentrations above 8%.

## Figures and Tables

**Table 1:** Chemical characterization of essential oils from eucalyptus species.

Compound	RIC	RIL	*Eucalyptus globulus*	*Eucalyptus citriodora*
			Composition (%)	
α-pinene	934	933	1.69	-
β-pinene	979	978	0.43	-
β-myrcene	990	989	0.50	-
p-cymene	1026	1025	3.79	-
Limonene	1030	1028	5.46	-
1,8-cineol	1034	1033	87.21	-
γ-terpinene	1060	1058	0.92	-
Isopulegol	1151	1150	-	4.90
Citronelal	1154	1153	-	90.26
Citronelol	1228	1228	-	4.84
Total identified	-	-	100	100

RIC = retention index calculated; RIL = retention index from the literature; composition (%) = percentage of compounds in essential oil.

**Table 2:** Determination of medium size, PDI, zeta potential, and pH.

Sample	Mean Size (nm)	PDI	Span	Zeta Potential (mV)	pH
NanoEE-Globulus	117.2 ± 0.9 b	0.214 ± 0.03 a	2.17± 0.30	−19.2 ± 0.5 a	6.6 ± 0.17 a
NanoEE-Citriodora	168.8 ± 1.2 a	0.209 ± 0.001 a	1.77 ± 0.21	−33.8 ± 1.2 b	6.8 ± 0.02 a

Results are expressed as mean ± standard deviation (*n* = 3). Different letters indicate a significant difference (*p* < 0.05) when analyzed by Tukey’s test in the column.

**Table 3:** Result for minimum inhibitory concentration and bacteriostatic effect on *Streptococcus mutans*.

	Bactericidal	Bacteriostatic
NanoEE-Globulus		
10%	-	-
8%	-	-
6%	-	-
4%	-	×
NanoEE-Citriodora		
10%	-	×
8%	-	×
6%	-	×
4%	-	-
Chlorhexidine		
0.12%	×	×

Caption: × indicates a positive result for microbiological inhibition or control; - indicates no control by the nanoemulsion, resulting in microbial growth.

## Data Availability

The original contribution presented in the study are included in the article, further inquiries can be directed to the corresponding author.

## References

[1] Pitts N.B., Zero D.T., Marsh P.D., Ekstrand K., Weintraub J.A., Ramos-Gomez F., Tagami J., Twetman S., Tsakos G., Ismail A. (2017). Dental Caries. Nat. Rev. Dis. Primers.

[2] Machiulskiene V., Campus G., Carvalho J.C., Dige I., Ekstrand K.R., Jablonski-Momeni A., Maltz M., Manton D.J., Martignon S., Martinez-Mier E.A. (2020). Terminology of Dental Caries and Dental Caries Management: Consensus Report of a Workshop Organized by ORCA and Cariology Research Group of IADR. Caries Res..

[3] Vos T., Abajobir A.A., Abate K.H., Abbafati C., Abbas K.M., Abd-Allah F., Abdulkader R.S., Abdulle A.M., Abebo T.A., Abera S.F. (2017). Global, Regional, and National Incidence, Prevalence, and Years Lived with Disability for 328 Diseases and Injuries for 195 Countries, 1990–2016: A Systematic Analysis for the Global Burden of Disease Study 2016. Lancet.

[4] Nazir M.A. (2017). Prevalence of Periodontal Disease, Its Association with Systemic Diseases and Prevention. Int. J. Health Sci..

[5] Kassebaum N.J., Arora M., Barber R.M., Bhutta Z.A., Brown J., Carter A., Casey D.C., Charlson F.J., Coates M.M., Coggeshall M. (2016). Global, Regional, and National Disability-Adjusted Life-Years (DALYs) for 315 Diseases and Injuries and Healthy Life Expectancy (HALE), 1990–2015: A Systematic Analysis for the Global Burden of Disease Study 2015. Lancet.

[6] Sanz M., Beighton D., Curtis M.A., Cury J.A., Dige I., Dommisch H., Ellwood R., Giacaman R.A., Herrera D., Herzberg M.C. (2017). Role of Microbial Biofilms in the Maintenance of Oral Health and in the Development of Dental Caries and Periodontal Diseases. Consensus Report of Group 1 of the Joint EFP/ORCA Workshop on the Boundaries between Caries and Periodontal Disease. J. Clin. Periodontol..

[7] Socransky S.S., Haffajee A.D. (2002). Dental Biofilms: Difficult Therapeutic Targets. Periodontology 2000.

[8] Araujo M.W.B., Charles C.A., Weinstein R.B., McGuire J.A., Parikh-Das A.M., Du Q., Zhang J., Berlin J.A., Gunsolley J.C. (2015). Meta-Analysis of the Effect of an Essential Oil–Containing Mouthrinse on Gingivitis and Plaque. J. Am. Dent. Assoc..

[9] Takenaka S., Sotozono M., Ohkura N., Noiri Y. (2022). Evidence on the Use of Mouthwash for the Control of Supragingival Biofilm and Its Potential Adverse Effects. Antibiotics.

[10] Sreenivasan P., Gaffar A. (2002). Antiplaque Biocides and Bacterial Resistance: A Review. J. Clin. Periodontol..

[11] Kalemba D., Kunicka A. (2003). Antibacterial and Antifungal Properties of Essential Oils. Curr. Med. Chem..

[12] Regnault-Roger C., Vincent C., Arnason J.T. (2012). Essential Oils in Insect Control: Low-Risk Products in a High-Stakes World. Annu. Rev. Entomol..

[13] Dhakad A.K., Pandey V.V., Beg S., Rawat J.M., Singh A. (2018). Biological, Medicinal and Toxicological Significance of *Eucalyptus* Leaf Essential Oil: A Review. J. Sci. Food Agric..

[14] Mahyari S., Mahyari B., Emami S.A., Malaekeh-Nikouei B., Jahanbakhsh S.P., Sahebkar A., Mohammadpour A.H. (2016). Evaluation of the Efficacy of a Polyherbal Mouthwash Containing Zingiber Officinale, *Rosmarinus officinalis* and Calendula Officinalis Extracts in Patients with Gingivitis: A Randomized Double-Blind Placebo-Controlled Trial. Complement. Ther. Clin. Pract..

[15] Stoeken J.E., Paraskevas S., Van Der Weijden G.A. (2007). The Long-Term Effect of a Mouthrinse Containing Essential Oils on Dental Plaque and Gingivitis: A Systematic Review. J. Periodontol..

[16] Nehavarshini V., Unnikrishnan S., Ramalingam K. (2023). Exploring the Potential of a Herbal Nanoemulsion as an Antimicrobial Mouthwash. Appl. Biochem. Biotechnol..

[17] Mostafa N.M. (2018). Antibacterial Activity of Ginger (Zingiber Officinale) Leaves Essential Oil Nanoemulsion against the Cariogenic Streptococcus Mutans. J. App Pharm. Sci..

[18] Horváth B., Balázs V.L., Varga A., Böszörményi A., Kocsis B., Horváth G., Széchenyi A. (2019). Preparation, Characterisation and Microbiological Examination of Pickering Nano-Emulsions Containing Essential Oils, and Their Effect on *Streptococcus mutans* Biofilm Treatment. Sci. Rep..

[19] Ostertag F., Weiss J., McClements D.J. (2012). Low-Energy Formation of Edible Nanoemulsions: Factors Influencing Droplet Size Produced by Emulsion Phase Inversion. J. Colloid Interface Sci..

[20] Jaiswal M., Dudhe R., Sharma P.K. (2015). Nanoemulsion: An Advanced Mode of Drug Delivery System. 3 Biotech.

[21] Linstrom P. (1997). NIST Chemistry WebBook, NIST Standard Reference Database 69. https://webbook.nist.gov/chemistry/.

[22] Van Den Dool H., Kratz P.D. (1963). A Generalization of the Retention Index System Including Linear Temperature Programmed Gas—Liquid Partition Chromatography. J. Chromatogr. A.

[23] Quatrin P.M., Verdi C.M., De Souza M.E., De Godoi S.N., Klein B., Gundel A., Wagner R., De Almeida Vaucher R., Ourique A.F., Santos R.C.V. (2017). Antimicrobial and Antibiofilm Activities of Nanoemulsions Containing *Eucalyptus globulus* Oil against *Pseudomonas aeruginosa* and *Candida* Spp.. Microb. Pathog..

[24] Cockerill F.R., Clinical and Laboratory Standards Institute, Clinical and Laboratory Standards Institute (2012). Methods for Dilution Antimicrobial Susceptibility Tests for Bacteria That Grow Aerobically: Approved Standard.

[25] Cimino C., Maurel O.M., Musumeci T., Bonaccorso A., Drago F., Souto E.M.B., Pignatello R., Carbone C. (2021). Essential Oils: Pharmaceutical Applications and Encapsulation Strategies into Lipid-Based Delivery Systems. Pharmaceutics.

[26] Gonçalves da Rosa C., Zapelini de Melo A.P., Sganzerla W.G., Machado M.H., Nunes M.R., Vinicius de Oliveira Brisola Maciel M., Bertoldi F.C., Manique Barreto P.L. (2020). Application in Situ of Zein Nanocapsules Loaded with Origanum Vulgare Linneus and Thymus Vulgaris as a Preservative in Bread. Food Hydrocoll..

[27] Rosso A., Lollo G., Chevalier Y., Troung N., Bordes C., Bourgeois S., Maniti O., Granjon T., Dugas P.-Y., Urbaniak S. (2020). Development and Structural Characterization of a Novel Nanoemulsion for Oral Drug Delivery. Colloids Surf. A Physicochem. Eng. Asp..

[28] Souto E.B., Cano A., Martins-Gomes C., Coutinho T.E., Zielińska A., Silva A.M. (2022). Microemulsions and Nanoemulsions in Skin Drug Delivery. Bioengineering.

[29] Nunes M.R., Da Rosa C.G., De Borba J.R., Dos Santos G.M., Ferreira A.L., Barreto P.L.M., Jana S., Jana S. (2022). Zein Nanoparticles: Bioactive Compounds and Controlled Delivery. Nanoengineering of Biomaterials.

[30] De Melo A.P.Z., Da Rosa C.G., Sganzerla W.G., Nunes M.R., Noronha C.M., Brisola Maciel M.V.D.O., Villetti M.A., Bertoldi F.C., Barreto P.L.M. (2019). Syntesis and Characterization of Zein Nanoparticles Loaded with Essential Oil of *Ocimum gratissimum* and *Pimenta racemosa*. Mater. Res. Express.

[31] Sganzerla W.G., Castro L.E.N., Da Rosa C.G., Almeida A.D.R., Maciel-Silva F.W., Kempe P.R.G., De Oliveira A.L.R., Forster-Carneiro T., Bertoldi F.C., Barreto P.L.M. (2023). Production of Nanocomposite Films Functionalized with Silver Nanoparticles Bioreduced with Rosemary (*Rosmarinus officinalis* L.) Essential Oil. J. Agric. Food Res..

[32] Jummes B., Sganzerla W.G., Da Rosa C.G., Noronha C.M., Nunes M.R., Bertoldi F.C., Barreto P.L.M. (2020). Antioxidant and Antimicrobial Poly-ε-Caprolactone Nanoparticles Loaded with Cymbopogon martinii Essential Oil. Biocatal. Agric. Biotechnol..

[33] Maciel M.V.D.O.B., Da Rosa C.G., Almeida A.D.R., Nunes M.R., Noronha C.M., Jummes B., Martelli S.M., Bertoldi F.C., Barreto P.L.M. (2021). Thymol Loaded Zein Microparticles Obtained by Spray-Drying: Physical-Chemical Characterization. Biocatal. Agric. Biotechnol..

[34] De Melo A.P.Z., Da Rosa C.G., Noronha C.M., Machado M.H., Sganzerla W.G., Bellinati N.V.D.C., Nunes M.R., Verruck S., Prudêncio E.S., Barreto P.L.M. (2021). Nanoencapsulation of Vitamin D3 and Fortification in an Experimental Jelly Model of Acca Sellowiana: Bioaccessibility in a Simulated Gastrointestinal System. LWT.

[35] Noronha C.M., De Carvalho S.M., Lino R.C., Barreto P.L.M. (2014). Characterization of Antioxidant Methylcellulose Film Incorporated with α-Tocopherol Nanocapsules. Food Chem..

[36] Lino R.C., De Carvalho S.M., Noronha C.M., Sganzerla W.G., Da Rosa C.G., Nunes M.R., D’Avila R.F., Zambiazi R.C., Barreto P.L.M. (2022). Production of Methylcellulose Films Functionalized with Poly-ε-Caprolactone Nanocapsules Entrapped β-Carotene for Food Packaging Application. Food Res. Int..

[37] Nunes M.R., Agostinetto L., Da Rosa C.G., Sganzerla W.G., Pires M.F., Munaretto G.A., Rosar C.R., Bertoldi F.C., Barreto P.L.M., Veeck A.P.D.L. (2024). Application of Nanoparticles Entrapped Orange Essential Oil to Inhibit the Incidence of Phytopathogenic Fungi during Storage of Agroecological Maize Seeds. Food Res. Int..

[38] Elangovan S., Mudgil P. (2023). Antibacterial Properties of Eucalyptus Globulus Essential Oil against MRSA: A Systematic Review. Antibiotics.

[39] Santos B., Farias J.H.A., Simões M.M., Medeiros M.A.A., Alves M.S., Diniz A.F., Soares A.P.O., Cavalcante A.P.T.M., Silva B.J.N., Almeida J.C.S. (2024). Evaluation of the Antimicrobial Activity of *Eucalyptus radiata* Essential Oil against *Escherichia coli* Strains Isolated from Meat Products. Braz. J. Biol..

[40] Harkat-Madouri L., Asma B., Madani K., Bey-Ould Si Said Z., Rigou P., Grenier D., Allalou H., Remini H., Adjaoud A., Boulekbache-Makhlouf L. (2015). Chemical Composition, Antibacterial and Antioxidant Activities of Essential Oil of Eucalyptus Globulus from Algeria. Ind. Crops Prod..

[41] Palmas L., Aroffu M., Petretto G.L., Escribano-Ferrer E., Díez-Sales O., Usach I., Peris J.-E., Marongiu F., Ghavam M., Fais S. (2020). Entrapment of Citrus Limon Var. Pompia Essential Oil or Pure Citral in Liposomes Tailored as Mouthwash for the Treatment of Oral Cavity Diseases. Pharmaceuticals.

[42] Santana Neto M.C., Costa M.L.V.D.A., Fialho P.H.D.S., Lopes G.L.N., Figueiredo K.A., Pinheiro I.M., De Lima S.G., Nunes R.D.S., Quelemes P.V., Carvalho A.L.M. (2020). Development of Chlorhexidine Digluconate and Lippia Sidoides Essential Oil Loaded in Microemulsion for Disinfection of Dental Root Canals: Substantivity Profile and Antimicrobial Activity. AAPS PharmSciTech.

[43] Horstmann Risso N., Stopiglia C.D., Oliveira M.T., Haas S.E., Ramos Maciel T., Reginatto Lazzari N., Kelmer E.L., Pinto Vilela J.A., Beckmann D.V. (2020). Chlorhexidine Nanoemulsion: A New Antiseptic Formulation. Int. J. Nanomed..

[44] Karnjana K., Jewboonchu J., Niyomtham N., Tangngamsakul P., Bunluepuech K., Goodla L., Mordmuang A. (2023). The Potency of Herbal Extracts and Its Green Synthesized Nanoparticle Formulation as Antibacterial Agents against *Streptococcus mutans* Associated Biofilms. Biotechnol. Rep..

[45] Toumba K.J., Twetman S., Splieth C., Parnell C., Van Loveren C., Lygidakis N.A. (2019). Guidelines on the Use of Fluoride for Caries Prevention in Children: An Updated EAPD Policy Document. Eur. Arch. Paediatr. Dent..

[46] Featherstone J.D.B. (1999). Prevention and Reversal of Dental Caries: Role of Low Level Fluoride. Comm. Dent. Oral Epid..

[47] Marquis R.E. (1995). Antimicrobial Actions of Fluoride for Oral Bacteria. Can. J. Microbiol..

[48] Goldbeck J.C., Do Nascimento J.E., Jacob R.G., Fiorentini Â.M., Da Silva W.P. (2014). Bioactivity of Essential Oils from *Eucalyptus globulus* and *Eucalyptus urograndis* against Planktonic Cells and Biofilms of *Streptococcus mutans*. Ind. Crops Prod..

[49] Salem N., Kefi S., Tabben O., Ayed A., Jallouli S., Feres N., Hammami M., Khammassi S., Hrigua I., Nefisi S. (2018). Variation in chemical composition of *Eucalyptus globulus* essential oil under phenological stages and evidence synergism with antimicrobial standards. Ind. Crops Prod.

[50] Choi J.E., Waddell J.N., Lyons K.M., Kieser J.A. (2016). Intraoral pH and Temperature during Sleep with and without Mouth Breathing. J. Oral. Rehabil..

